# Function and 3D Structure of the *N*-Glycans on Glycoproteins

**DOI:** 10.3390/ijms13078398

**Published:** 2012-07-06

**Authors:** Masamichi Nagae, Yoshiki Yamaguchi

**Affiliations:** Structural Glycobiology Team, RIKEN, Advanced Science Institute, 2-1 Hirosawa, Wako-City, Saitama 351-0198, Japan; E-Mail: mnagae@riken.jp

**Keywords:** glycoprotein, glycoform, *N*-glycan, protein crystallography

## Abstract

Glycosylation is one of the most common post-translational modifications in eukaryotic cells and plays important roles in many biological processes, such as the immune response and protein quality control systems. It has been notoriously difficult to study glycoproteins by X-ray crystallography since the glycan moieties usually have a heterogeneous chemical structure and conformation, and are often mobile. Nonetheless, recent technical advances in glycoprotein crystallography have accelerated the accumulation of 3D structural information. Statistical analysis of “snapshots” of glycoproteins can provide clues to understanding their structural and dynamic aspects. In this review, we provide an overview of crystallographic analyses of glycoproteins, in which electron density of the glycan moiety is clearly observed. These well-defined *N*-glycan structures are in most cases attributed to carbohydrate-protein and/or carbohydrate-carbohydrate interactions and may function as “molecular glue” to help stabilize inter- and intra-molecular interactions. However, the more mobile *N*-glycans on cell surface receptors, the electron density of which is usually missing on X-ray crystallography, seem to guide the partner ligand to its binding site and prevent irregular protein aggregation by covering oligomerization sites away from the ligand-binding site.

## 1. Introduction

Glycosylation is a common and highly diverse modification of proteins and occurs during or after protein synthesis [1]. More than 50% of eukaryotic proteins are glycosylated [2]. Glycosylation profoundly alters the behavior of proteins, making them more soluble, protecting them from proteolysis, covering antigenic sites, and altering the orientation of proteins on cell surfaces. The importance of protein glycosylation is becoming widely recognized through studies on protein localization and trafficking, biological half-life as well as investigations of cell-cell interactions. In almost all glycoproteins, the carbohydrate units are attached to the protein backbone either by *N*- or *O*-glycosidic bonds or both. *N*-glycans are covalently attached to proteins at the amide of asparagine (Asn) residues, forming an *N*-glycosidic bond. The consensus sequence for *N*-glycosylation, called a sequon, is Asn-X-Ser/Thr, where X can be any amino acid except proline. In *O*-glycosylation, the glycan is attached to the side chains of serine or threonine residues. Unlike *N*-linked glycosylation, no consensus sequence defining an *O*-linked glycosylation site has been reported.

**Figure 1:**
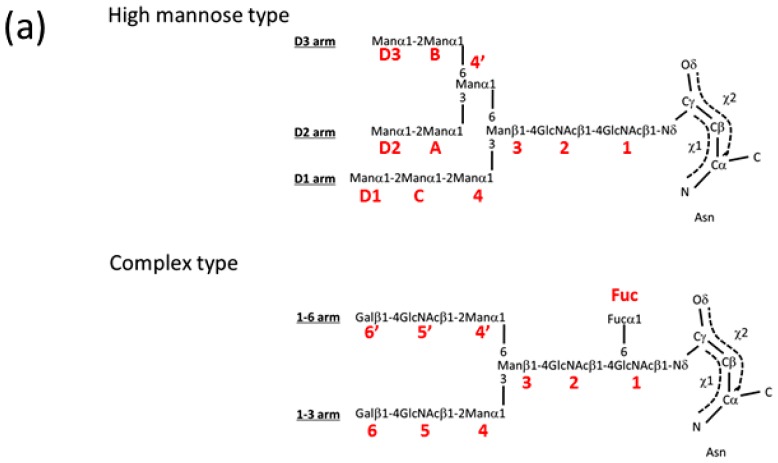

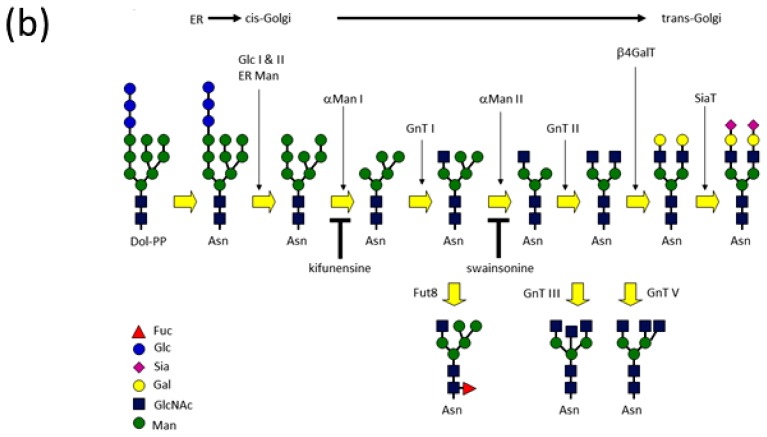
(**a**) Representative chemical structures of high-mannose and complex-type *N*-glycans. (**b**) *N*-glycan processing pathways in mammalian cells. The enzymes and structures of intermediate *N*-glycans are shown. Glc I, α-glucosidase I; Glc II, α-glucosidase II; ER Man, ER α-mannosidase; α-ManI, α-mannosidase I; GnT I, β-*N*-acetylglucosaminyltransferase I; α-ManII, α-mannosidase II; GnT II, β-*N*-acetylglucosaminyltransferase II; β4GalT, β-1,4-galactosyltransferase; SiaT, sialyltransferase; GnT III, β-*N*-acetylglucosaminyltransferase III; GnT V, β-*N*-acetylglucosaminyltransferase V; and α1,6-fucosyltransferase, Fut8.

Human *N*-glycan is typically composed of *N*-acetyl-d-glucosamine (GlcNAc), d-mannose (Man), d-galactose (Gal), sialic acid (general name for *N*-acetylneuraminic acid, which can be abbreviated to Sia or Neu5Ac), d-glucose (Glc) and l-fucose (Fuc) residues. *N*-glycan is classified into three groups: high-mannose type, hybrid type, and complex type. Representative chemical structures of high-mannose (Man_9_GlcNAc_2_) and biantennary complex-type (Gal_2_GlcNAc_2_Man_3_GlcNAc_2_) glycans are shown in Figure 1a. Both types contain Man_3_GlcNAc_2_ core structures. In high-mannose type glycan, Manα1-2Manα1-2Man, Manα1-2Manα1-3Man, and Manα1-2Manα1-6Man chains are designated the D1, D2 and D3 arms, respectively. In biantennary complex-type glycan, the α1-3, and α1-6 branched oligosaccharide chains (Galβ1-4GlcNAcβ1-2Man) are termed α1-3 and α1-6 arms, respectively. *N*-glycan precursor (Glc_3_Man_9_GlcNAc_2_) is assembled on a lipid carrier, dolichylpyrophosphate (Dol-PP), and is transferred onto a polypeptide chain by oligosaccharyltransferase (OST) in the endoplasmic reticulum (ER) lumen (Figure 1b). The attached *N*-glycan is sequentially processed by various glycosyl-hydrolases and -transferases. Three glucose and one mannose residues are immediately removed by glucosidase I, II and ER α-mannosidase. Removal of glucose residues is closely related to the folding of glycoproteins. Further processing of *N*-glycans occurs along the secretory pathway as the properly folded glycoprotein moves through the Golgi apparatus to its final destination. The initial stage of the pathway consists of several trimming steps by α-mannosidase I that generate a key intermediate, Man_5_GlcNAc_2_. In the Golgi apparatus, the conversion of high-mannose type to hybrid-type glycan is initiated by the action of *N*-acetylglucosaminyl transferase I (GnT I), which transfers GlcNAc, in a β1-2 linkage, to the α1-3 arm mannose residue of Man_5_GlcNAc_2_ substrate. Hybrid-type glycan is further remodeled by a series of enzymes to yield complex-type *N*-glycans. There are several pathways which increase the heterogeneity of *N*-glycans, including *N*-acetylglucosaminyltransferase III (GnT III), V (GnT V), and α1,6-fucosyltransferase (Fut8) (Figure 1b). GnT III catalyzes the addition of GlcNAc via a β1-4 linkage to the β-Man of the mannosyl core of *N*-glycans. This GlcNAc is designated as “bisecting GlcNAc” and is involved in the suppression of cancer metastasis [3]. GnT V catalyzes the addition of a β1-6 linked GlcNAc unit to α1-6 linked Man of the trimannosyl core of *N*-linked glycans to form tri- or tetra-antenary branches [4]. Fut8 introduces a fucose residue onto the innermost GlcNAc of the *N*-linked biantennary complex-type oligosaccharides via an α1-6 linkage [5] (Figure 1b). This reaction is denoted as “core-fucosylation” and the Fuc residue is termed “core-fucose”. Each glycosyltransferase has unique substrate specificity that determines the *N*-glycan structure. For example, the action of GnT V and Fut8 is inhibited by the presence of a bisecting GlcNAc residue. Through these modifications, complex-type glycan is often branched and contains a trimannosyl core, several GlcNAc, Gal, sialic acid and Fuc residues, resulting in a high-level of complexity (Figure 1b).

3D structural information on glycoproteins helps in the understanding of *N*-glycan function. However, the inherent flexibility of glycan hampers X-ray crystallographic analysis. Actually, diffraction quality crystals of glycoproteins are normally only obtained following either the abolition of the glycosylation site by site-directed mutagenesis or enzymatic deglycosylation treatment [6–8]. The number of glycoproteins resolved by X-ray or NMR represents less than 3% of the total number of reported 3D structures in the Protein Data Bank (PDB) [9]. However, significant improvements in experimental methods have led to an increase in the number of solved glycoprotein structures with well-defined carbohydrate residues. Various methodologies using mammalian expression systems have been developed for the production of large amounts of homogeneous glycoproteins. Chinese hamster ovary (CHO)-lec 3.2.8.1 cells [10] and human embryonic kidney (HEK) 293S GnT I deficient cells [11] are suitable for producing glycoproteins for crystallization. Since both cell lines lack GnT I activity, uniform glycoprotein can be produced with the truncated *N*-linked oligosaccharide, Man_5_GlcNAc_2_ (Figure 1b). Moreover, co-cultivation of mammalian cells with a *N*-glycosylation inhibitor, kifunensine or swainsonine, reduces the chemical heterogeneity of the product glycoform and can be readily deglycosylated with endoglycosidase (Figure 1b, [12]). Other eukaryotic expression systems, such as yeast and insect cells, are also available for producing homogeneous protein for crystallography. However, in fungal and insect expression systems, mature *N*-linked glycan is generally of the heterogeneous high-mannose type, whereas human *N*-glycans are mainly hybrid or complex types. Since glycan structure depends on the expression cell type, the relationship between glycoform and its physiological function needs careful inspection.

Previous statistical analyses of the available X-ray diffraction data on oligosaccharides [9,13,14] identified the energetically-favorable conformations for individual sugar linkages. GLYCOSCIENCES.de web portal [15] and the Glycoconjugate Data Bank [16] offer convenient ways to search for carbohydrate structures in the PDB. In this review, we introduce several examples in which the glycans affect intra- and inter-molecular interactions. The glycans are often stabilized to assume highly ordered structures via extensive protein-carbohydrate or carbohydrate-carbohydrate interactions. We also introduce several cell-surface glycoprotein structures where the glycans seem to assist in proper ligand recognition and to prevent protein aggregation, although many of these glycans have disordered structures.

## 2. Conserved Glycan Mediates Inter-Subunit Interactions of Proteins (Fc Fragment and Influenza Neuraminidase)

### 2.1. *N*-Glycan on Fc Fragment Affects Intra- and Inter-Molecular Interactions

#### 2.1.1. Overview of *N*-Glycan of Fc Fragment

**Figure 2:**
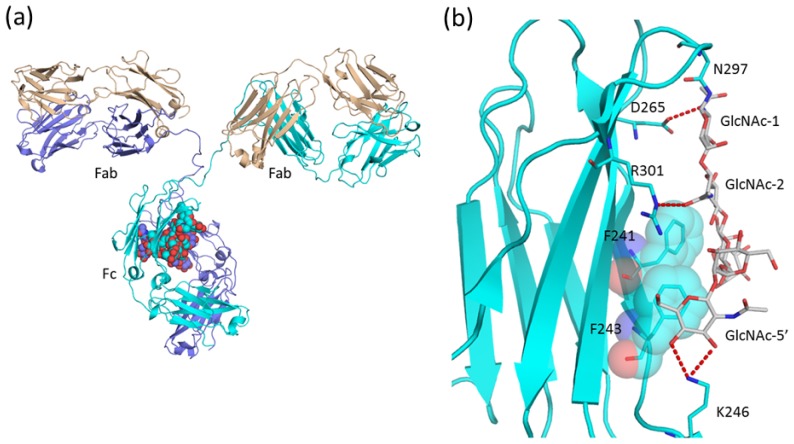
(**a**) Overall structure of immunoglobulin G (PDB code; 1igt) is shown in a ribbon model. One light and two heavy chains are shown in beige, blue and cyan, respectively. Carbohydrate residues attached on the Fc region are shown in sphere models. (**b**) Close-up view of Asn297 attached glycan of human IgG1 Fc (PDB code; 2dts). Carbohydrate moiety and amino acid residues which interact with *N*-glycan are shown in the rod model. Hydrogen bonds between protein and carbohydrate are shown as red dotted lines.

Immunoglobulins are Y-shaped glycoproteins that participate in the adaptive component of the immune system which is directed against extracellular pathogens. The structural diversity of their antigen binding sites allows them to bind specifically to millions of structurally unique molecules. Immunoglobulin G (IgG) consists of two light and two heavy chains and comprises three independent parts connected through a flexible linker or hinge (Figure 2a). Two of these, the Fab fragments, are identical in structure, each with an antigen-specific binding site. The third one, the Fc fragment, has a highly conserved structure even between different isotypes [17–20]. It has antibody effector functions such as antibody-dependent cellular cytotoxicity (ADCC) and complement-dependent cytotoxicity (CDC) through its interaction either with lymphocyte receptors (Fc receptors, FcγRs) on effector cells such as natural killer cells or with the C1q component of complement. The Fc fragment is a dimer and displays a horseshoe-like arrangement of two antiparallel β-sandwich domains, named C_H_2 and C_H_3, connected by a short flexible linker (Figure 2a). Interaction between subunits is mainly through the pair of C_H_3 domains. These two domains form a compact, non-covalent dimer with a buried surface area of ~1,000 Å^2^. A biantennary complex-type glycan is attached onto each Asn297 in the C_H_2 domains [17]. Characteristics of Fc *N*-glycosylation include a low incidence of monosialylation, no disialylation, little bisecting GlcNAc, a high incidence of core fucose and heterogeneity of galactose residues [21]. The particular glycoform of the Fc fragment impacts on its physiological function. Removal of fucose is known to enhance ADCC activity both *in vitro* [22] and *in vivo* [23]. Moreover, the galactose content of human IgG-Fc correlates inversely with disease progression in rheumatoid arthritis and other auto-immune diseases [24]. The anti-inflammatory activity of intravenous Ig (IVIG) can be recapitulated with a fully recombinant preparation of appropriately sialylated IgG Fc fragments [25]. Thus, manipulation of Asn297 glycan structures has emerged as a strategy to modulate effector functions of therapeutic antibodies [26,27].

Pioneering X-ray crystallographic studies of the isolated human IgG1 Fc domain [17] and rabbit IgG [28] have shown that the two conserved *N*-glycan chains of the Fc are well defined, with the oligosaccharide bound to the surfaces of the C_H_2 domains. Since then, our structural knowledge of Fc fragments and their glycans have grown substantially. As of March 2012, there are more than 30 PDB entries. The structures containing glycosylated Fc fragments are summarized in Table 1. A representative structure of human IgG1 Fc and its *N*-glycan is shown in Figure 2b. The α1-6 arm of the *N*-glycan of Asn297 contacts two hydrophobic residues, Phe241 and Phe243, and makes several hydrogen bonds with Lys246, Asp265, and Arg301 (Figure 2b). A core Fuc is located in the vicinity of Tyr296 and indirectly affects the hydration mode of Tyr296 [29]. In contrast, Tyr313, which corresponds to Tyr296 in human IgG1, makes direct hydrogen bonds with a core Fuc residue in the mouse IgG2a structure [19].

**Figure 3:**
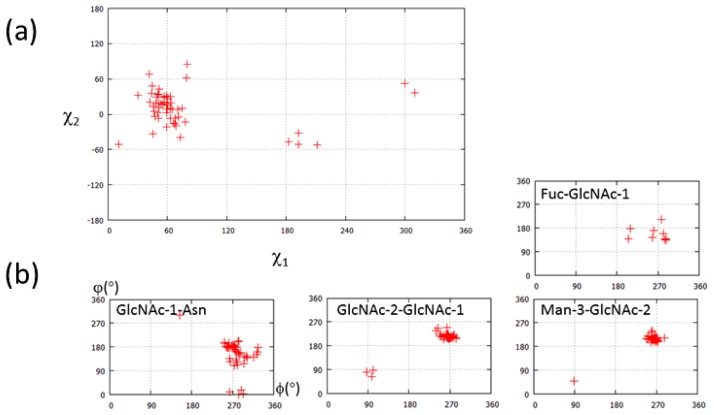

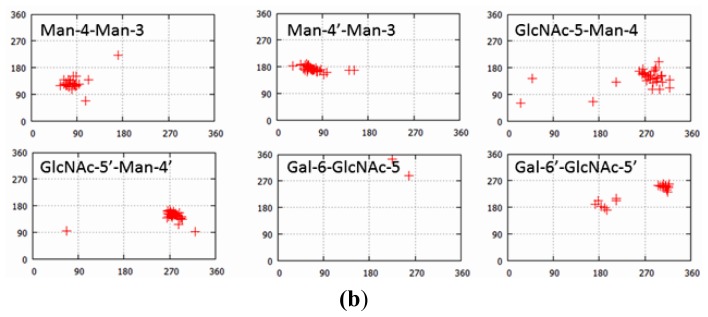
(**a**) The side-chain torsions of Asn297 of the Fc fragment. Torsion angles of the Asn297 side chain are measured by MolProbity [52]. We excluded Fc with high-mannose type glycan (PDB code 2wah) from this inspection, since this structure contains high-mannose type glycans. (**b**) Comparison of glycosidic torsions of *N*-glycan attached on Asn297 of Fc fragment. Dihedral angles of each linkage are calculated with CARP [15]. The vertical and horizontal axes indicate *ϕ* and *φ* angles, respectively. The residues with errors are carefully excluded from this analysis. In many cases, a β1-6 linkage is erroneously used between core Fuc and GlcNAc instead of an α1-6 bond [53]. Eight entries are plotted in Fuc-GlcNAc-1 (PDB code; 1h3w, 3ave, 3d6g, 2rgs, chain-A in 1e4k, and chain-B in 3sgj).

Previous comparative conformational analysis has demonstrated that both glycosylated and non-glycosylated Asn side chains exhibit *χ*1 (N-Cα-Cβ-Cγ) torsion angles of −60°, 60°, 180°, corresponding to the *g*−, *g*+ and *t* conformers, respectively. The *χ*2 (Cα-Cβ-Cγ-Nδ) torsion angle shows a wide range centered at about 0°, but is nevertheless limited by glycosylation. In the case of glycosylated Asn side chains, the *g*- conformer is most preferred and the *g*^+^ conformer rarest in both glycosylated and non-glycosylated Asn residues [9,48]. The conformation of 56 Fc *N*-glycans is statistically analyzed in Figure 3, from the 29 different Fc structures listed in Table 1. The side chain torsion angles, *χ*1 and *χ*2, of Asn297 are plotted in Figure 3a. In the case of Fc structures, the *χ*1 torsion angle of Asn297 exhibits a marked preference for ~60°, corresponding to the *g*^+^ conformer. The torsion angles, *φ* and *ψ*, for all glycosidic linkages are plotted in Figure 3b. All the dihedral angles of the glycosidic linkages are within an acceptable region compared with other glycoproteins [48]. The dihedral angle distribution of the α1-6 arms appears to be more restricted than that of the α1-3 arms (Man-4-Man-3, Man-4′-Man-3, GlcNAc-5-Man-4, and GlcNAc-5′-Man-4′ in Figure 3b). This difference derives from the fact that the α1-6 arms of the glycan interact with the first two strands of the C_H_2 domains. Solution NMR analyses of the Fc indicate that the α1-6 arm is completely immobilized through interactions with the protein surface, whereas the α1-3 mannose terminus is more dynamic [49,50]. On the other hand, spin relaxation NMR studies indicate that Fc glycan could exhibit a dynamic conformational state as well as the fixed state as seen in the crystal structure [51].

#### 2.1.2. Glycoform Affects the Relative Interdomain Angles of the Fc Fragment

Glycan structure can potentially affect the overall structure of a glycoprotein. The influence of glycoform on the conformation of the Fc fragment has been extensively investigated. Two papers report on the relationship between glycoform and the interdomain angles of the C_H_2-C_H_3 domains.

In the first report, the influence of glycoform on the structure and function of IgG Fc was assessed by sequential exo-glycosidase treatment [31]. Krapp *et al.* solved the crystal structures of human IgG1 Fc of four glycoforms bearing consecutively truncated oligosaccharides (PDB code; 1h3t, 1h3u, 1h3x, 1h3v and 1h3w). Removal of the terminal GlcNAc as well as the mannose residues causes the largest conformational change in both the oligosaccharide and in the polypeptide loop containing the *N*-glycosylation site. The conformational change in the C_H_2 domain affects the interface between the IgG-Fc fragments and the FcγRs. Moreover, removal of the sugar residues permits the mutual approach of the C_H_2 domains and the generation of a closed conformation. This contrasts with the open conformation of fully galactosylated IgG Fc, which may be optimal for FcγR binding. Solution NMR analysis of a series of Fc glycoforms also indicates that the carbohydrate moieties are required for maintaining the structural integrity of the FcγR binding site [54].

**Figure 4:**
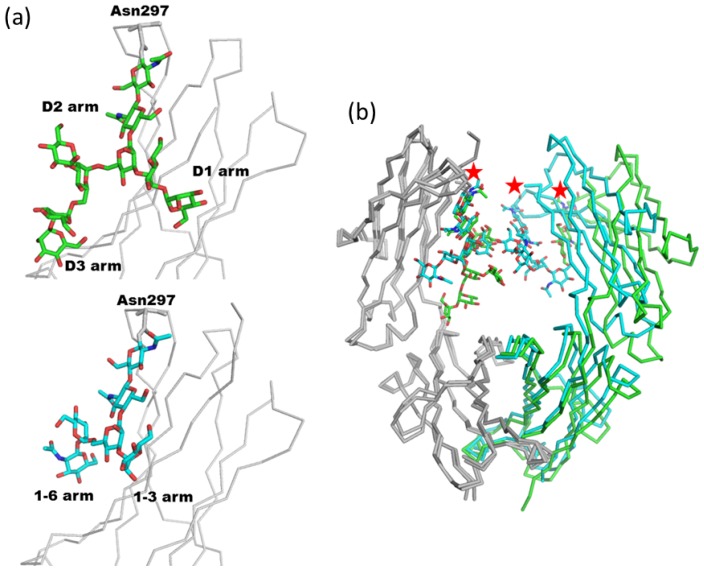
(**a**) Structural comparison between high-mannose glycan (PDB code; 2wah, green) and complex-type glycan (PDB code; 2dts, cyan). (**b**) Structural superposition between high-mannose type Fc fragment (green) and complex-type Fc fragment (cyan). Protein molecules and carbohydrate chains are shown in wire and stick models, respectively. The positions of Asn297 are indicated by red asterisks. Structural superposition of crystal structures were performed by the program SUPERPOSE [56].

In the second report, Crispin and co-workers solved the crystal structure of a recombinant human IgG1 Fc fragment designed to possess high-mannose type glycan at Asn297 [30]. Recombinant Fc was transiently expressed using HEK 293T cells in the presence of the α-mannosidase I inhibitor, kifunensine (Figure 1b). The glycan structure was confirmed to be Man_9_GlcNAc_2_ by MALDI-TOF-MS and the crystal structure of the glycoform was solved. The electron density map of the high-mannose glycan is quite asymmetric. Extensive branched density is observed in chain A, assigned as Man_7_GlcNAc_2_. On the other hand, poor electron density is found in chain B corresponding to three reducing terminal saccharide residues (Man_1_GlcNAc_2_). The overall structure of the high-mannose glycan is similar to that of a complex type. In Figure 4a, there is a structural comparison between high-mannose type (chain A of 2wah) and complex-type glycans (PDB code; 2dts). The *χ*1 and *χ*2 angles of the side chain of Asn297 are essentially identical to those of other Fc fragments possessing complex-type glycans. Moreover, the dihedral angles of the glycosidic linkages in a pentasaccharide Man_3_GlcNAc_2_ core of both glycans are also similar (Figure 4a). However, the α1-6 branches (D2 and D3 arms) of the high-mannose type occupy different positions compared with complex-type glycans. This is due to the difference between the α1-6 and β1-2 glycosidic bonds. Structural superposition of two Fc fragments reveals that the attachment of high-mannose type *N*-glycans opens the interdomain cavity between the C_H_2 domains (Figure 4b). This structural difference between the two glycoforms might explain the evidence that recombinant monoclonal antibody with human oligomannose-type glycans display enhanced ADCC, together with reduced complement activation through C1q binding [55]. It should be noted that the expression construct of the recombinant Fc fragment lacks the cysteine residues which form a disulfide bond in the hinge region. Further studies are needed to fully reveal the relationship between glycoform and structure.

#### 2.1.3. Intra-molecular Carbohydrate-Carbohydrate Interaction

**Figure 5:**
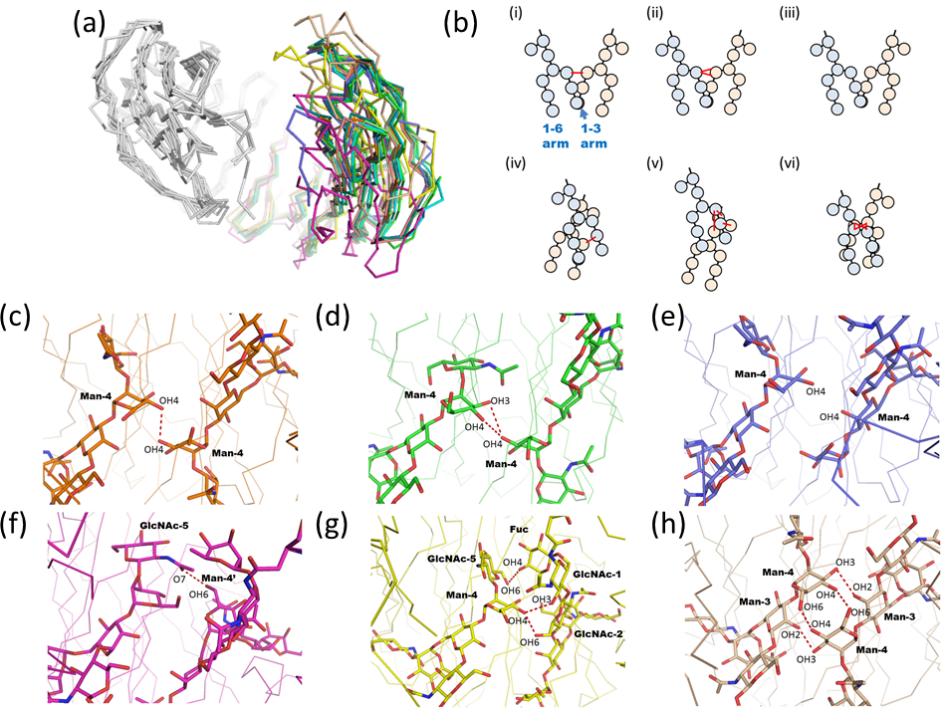
(**a**) Structural superposition of 10 Fc fragment structures (1fc1; green, 1h3v; cyan, 1h3y; magenta, 1i1c; yellow, 2dts; pink, 1i1c; yellow, 2rgs; wheat, 2vuo; slate, 3ave; orange, 3do3; lime, 3fjt; deep teal). For four entries (PDB code; 1h3w, 3c2s, 2ql1, and 1l6x), the asymmetric units of these crystals contain only one heavy chain. Thus, the symmetry-related neighboring heavy chains were compensated for in this analysis. Structural superposition was performed by SUPERPOSE. (**b**) Schematic representation of six types of carbohydrate-carbohydrate interaction modes. *N*-glycans of two chains are shown in blue and pink. Hydrogen bonds are shown as red lines. (**c**)–(**h**) Close-up views of the interfaces of carbohydrate-carbohydrate interactions. Carbohydrate moiety is shown in the rod model. Hydrogen bonds between carbohydrates are shown as red dotted lines. The structural superimposition of four structures which have only one carbohydrate-carbohydrate interaction is shown in (c). Human IgG1 Fc fragment (PDB code; 1fc1) is shown in (d). The superimposition of two structures which have no interaction between glycans is shown in (e). Human IgG1 Fc in high salt condition (PDB code; 1h3y), rat IgG2a (PDB code; 1i1c), and mouse IgG2b Fc fragment (PDB code; 2rgs) are shown in (f), (g), and (h), respectively.

Direct C_H_2-C_H_2 interaction is accomplished mainly through the pair of *N*-glycans at Asn297. To analyze the intramolecular carbohydrate-carbohydrate interaction modes of Fc fragment, we compared 10 uncomplexed Fc fragment structures from Table 1; wild type human IgG1 Fc (PDB code; 1fc1, 1h3v, 1h3y, 2dts, 3ave, and 3do3), mutated human IgG1 Fc (PDB code; 3fjt), rat IgG2a Fc (PDB code; 1i1c), mouse IgG2b Fc (PDB code; 2rgs), and rabbit IgG Fc (PDB code; 2vuo). Structural superimposition among these 10 Fc structures reveals that the interdomain angles of the C_H_2-C_H_3 domains are highly variable (Figure 5a). The highly mobile domain angles result in a variety of carbohydrate-carbohydrate interaction modes as described below. The modes are classified into six types (i~vi), and a schematic representation is shown in Figure 5b. (i) In four of 10 Fc fragments (PDB code; 2dts, 3fjt, 3ave, and 3do3), only one hydrogen bond is found between the OH4 hydroxyl group of each α1-3 linked Man residue (Man-4) (Figure 5c). (ii) In the first reported human IgG1 Fc fragment structure (PDB code; 1fc1), the carbohydrate-carbohydrate interaction mode is slightly different. The OH4 hydroxyl group of the α1-3 linked Man (Man-4) asymmetrically interacts with both OH3 and OH4 of the counterpart α1-3 linked Man (Man-4) (Figure 5d). (iii) In two structures of human IgG1 Fc fragment (PDB code; 1h3v and 2vuo), the distances between the two OH4 hydroxyl groups of the two α1-3 linked Man (Man-4) residues are rather long for a direct hydrogen bond (Figure 5e). In (i) ~ (iii), the Fc structures superimpose well, whereas the remaining three Fc structures (PDB code; 1h3y, 1i1c, and 2rgs) are unique. (iv) The structure of the human IgG1 Fc fragment (PDB code; 1h3y) is completely different compared with other human wild type IgG1 Fc fragments (see magenta in Figure 5a). In this structure, one weak hydrogen bond is observed between O7 of GlcNAc in the α1-3 arm (GlcNAc-5) and OH6 hydroxyl group of the α1-6 linked Man (Man-4′) at a distance of 3.2 Å (Figure 5f). The human IgG1 Fc fragment (1h3y) was crystallized in a high salt concentration (2.0 M NaCl) while the other human wild type IgG1 Fc fragments were crystallized under lower ionic strength conditions (1fc1; 30 mM sodium chloride, 1h3v and 2dts; distilled water, 3ave; 20% butanediol, and 3do3; 0.2 M sodium chloride and 20% polyethylene glycol 3,350). The range of ionic strength conditions may contribute to the conformational differences (v) The interaction mode observed in rat IgG2a Fc (PDB code; 1i1c) is asymmetric. The α1-3 arm of one side contacts the core Fuc and the chitobiose of the other (Figure 5g). The O5 and OH6 of GlcNAc in the α1-3 arm (GlcNAc-5) interact with OH4 of the Fuc residue, whereas OH4 of the α1-3 linked Man (Man-4) makes hydrogen bonds with the hydroxyl groups of the chitobiose. (vi) In the crystal structure of mouse IgG2b Fc fragment (PDB code; 2rgs, [40]), four symmetric hydrogen bonds are found between the two oligosaccharide chains. The OH3 of the α1-3 linked Man (Man-4) makes hydrogen bonds with OH2 of its counterpart β-Man (Man-3), and likewise the OH4 with OH6 (Figure 5h).

The interdomain angle of the C_H_2-C_H_3 domains could be affected by the crystallization conditions, especially ionic strength [31]. Crystallographic analysis only provides static snapshots and gives no direct information on the dynamics of a glycosidic linkage or of polypeptide fluctuations. Thus, it is likely that the single static linkage conformation observed in a crystal does not directly reflect the average solution conformation. Nonetheless, the average conformation for a given linkage within a large set of static structures is likely to correspond well to the average solution conformation, and the distribution of static structures will give an indication of the flexibility of the linkage, as long as crystal packing forces do not impose systematic changes.

#### 2.1.4. Carbohydrate-Assisted Intermolecular Interaction (Neonatal Fc Receptor and Fcγ Receptor IIIa)

Carbohydrates attached to glycoproteins are often required for tight binding to partner proteins. In this section, two topics are introduced in which *N*-linked carbohydrates on receptors contribute to full Fc binding activity.

**Figure 6:**
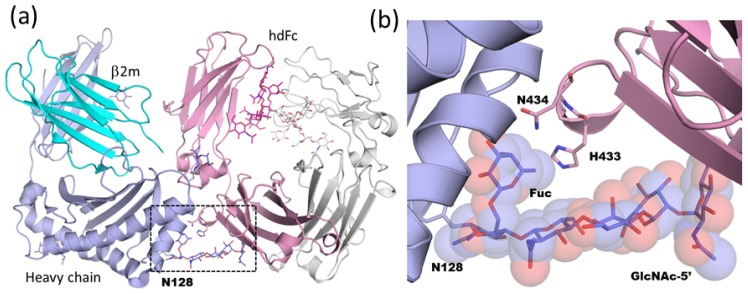

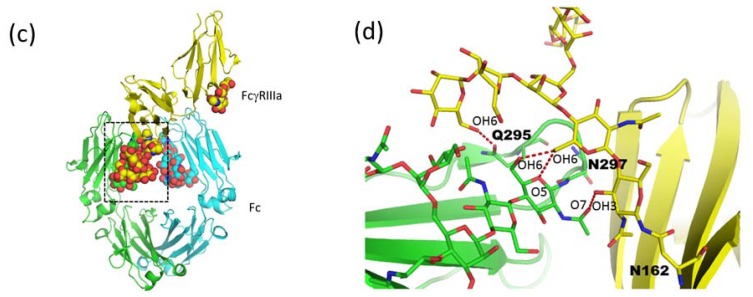
(**a**) Overall structure of neonatal Fc receptor (FcRn) in complex with heterodimeric Fc (hdFc) (PDB code; 1i1a). Heavy chain and soluble light chain β2-microglobulin (β2m) of FcRn are shown in slate and cyan, respectively. Proximal and distal Fc fragments of hdFc are shown in pink and white, respectively. The region delineated in black dotted lines is magnified in (b). (**b**) Close-up view of FcRn-hdFc complex. *N*-glycan attached at Asn128 of FcRn is shown in rod and semitransparent sphere model. (**c**) Overall structure of human Fc-glycosylated human Fcγ receptor IIIa (FcγRIIIa) complex (PDB code; 3sgk). Two chains of Fc fragment and FcγRIIIa are shown in green, cyan, and yellow, respectively. The region delineated in black dotted lines is magnified in (d). (**d**) Close-up view of carbohydrate-carbohydrate interaction in Fc-FcγRIIIa. Hydrogen bonds are shown as red dotted lines.

The neonatal Fc receptor (FcRn) transports maternal immunoglobulin G (IgG) across the neonatal intestine in rodents and across the placenta in humans, thereby conferring humoral immunity to the fetus or newborn against antigens encountered by the mother. FcRn binds IgG with nanomolar affinity at acidic pH (≤6.5) in the intracellular transport vesicles and releases IgG upon encountering the basic pH of the bloodstream (7.4). FcRn is a heterodimer and is composed of a soluble light chain β2-microglobulin and a membrane-bound heavy chain that includes three extracellular domains (α1~3), a single pass transmembrane domain, and a short cytoplasmic domain. FcRn interacts with the C_H_2-C_H_3 domain interface on each chain of the Fc homodimer [57]. The 2:2 interaction mode creates higher ordered structures, called “oligomeric ribbons”, and prohibits the growth of well-ordered co-crystals. To improve the crystal quality, Martin and co-workers designed a heterodimeric version of Fc that cannot bridge between FcRn molecules, since it contains only a single FcRn binding site [58], and solved the crystal structure of a FcRn ectodomain in complex with the heterodimeric Fc fragment at 2.8-Å resolution [18]. The heavy chain (α2) and β2-microglobulin domains of FcRn interact with the Fc C_H_2-C_H_3 interface (Figure 6a). The binding interface between FcRn and Fc spans a large surface area (buried surface area up to 1,870 Å^2^) and is highly complementary. The complex is stabilized by extensive hydrophobic and electrostatic interactions. Interestingly, the complex-type *N*-linked glycan attached on Asn128 of FcRn has a highly ordered structure and contributes 10–15% of the total buried surface area in the interface. The core Fuc residue and GlcNAc of the α1-3 arm (GlcNAc-5) tightly interact with the complementary surface of the binding partner (Figure 6b). This suggests that complex-type glycan, but not high-mannose type glycan, on FcRn is required for maximal Fc binding affinity. Actually, differential glycosylation of mouse FcRn could affect the receptor/ligand stoichiometry under non-equilibrium conditions [59].

The crystal structure of the Fc-FcRn complex reveals how the *N*-glycan on FcRn affects the binding affinity. In contrast, the *N*-glycan of IgG Fc strongly influences the interaction between IgG Fc and FcγR, and therapeutic antibodies may be modulated by selecting the appropriate glycoform. For example, removal of Fc core fucose selectively and significantly increases binding affinity to FcγRIII, thereby leading to enhanced cellular immune effector functions, such as ADCC. This may be especially relevant with respect to therapeutic anticancer antibodies [60,61], and has been the focus of research during the past decade. A crystal structure of glycosylated human IgG1 Fc fragment in complex with the unglycosylated extracellular domain of FcγRIII was first reported by Sondermann and co-workers [45]. One FcγRIII binds to the two halves of the Fc fragment and contacts residues in the C_H_2 domains and the hinge region. Complex formation significantly increases the angle between the two soluble FcγRIII domains and the Fc fragment is asymmetrically open. Recently, crystal structures of afucosylated human Fc in complex with glycosylated FcγRIIIa ectodomain were solved by two independent groups (Figure 6c) [46,47]. Although both groups produced the glycosylated FcγRIIIa ectodomain using mammalian expression systems, the glycan structures attached on Asn162 differ. Ferrara and colleagues report that the *N*-glycan structure is high-mannose type, while Mizushima *et al.* find asialylated complex type. The overall fold of the Fc-FcγRIIIa complexes where both proteins are glycosylated is very similar to that of the complexes where only the Fc protein is glycosylated. Clear electron density was obtained for both the Asn162-linked glycan of the receptor and the glycans linked to the Fc fragment. The carbohydrate attached on Asn162 shares a large interaction surface area (approximately 12% of the total interface area —145 Å^2^—in the case of PDB code; 3ay4) with the Fc formed by various polar, van der Waals, and hydrogen bond interactions. The receptor Asn162-carbohydrate interactions center on the Asn297-carbohydrate core of Fc chain A and its immediate vicinity (Figure 6d). Overall, a combination of direct or water-mediated carbohydrate-carbohydrate and carbohydrate-protein contacts are observed as part of the newly formed interaction between afucosylated Fc and the Asn162-glycosylated receptor.

Ferrara and colleagues also solved the crystal structure of fucosylated Fc in complex with glycosylated FcγRIIIa ectodomain. The core fucose linked to Fc is oriented towards the second GlcNAc (GlcNAc-2) of the chitobiose connected to Asn162 of FcγRIIIa and has to be accommodated in the interface between the interacting glycan chains. This steric rearrangement causes the movement of the whole oligosaccharide attached on Asn162 up to a maximum distance of 2.6 Å while almost no movement is observed in the case of afucosylated Fc. This rearrangement of the interaction network reduces the enthalpy contribution in the fucosylated Fc complex. It is noteworthy that even such subtle displacement of carbohydrate chains affects physiological activity, such as in ADCC [46].

### 2.2. High-Mannose Type Glycan on Group 2 Influenza Virus Neuraminidase

Influenza virus infection has been a major threat to public health throughout the world for centuries. Influenza types A and B are enveloped RNA viruses carrying two glycoproteins on their surface, hemagglutinin (HA) and neuraminidase (NA, acylneuraminyl hydrolase, EC 3.2.1.18). Influenza NA removes terminal α2-3 or α2-6 linked sialic acid residues from carbohydrate moieties on cell surface glycoconjugates and is thought to thereby facilitate virus release and infection of another cell. Inhibition of NA delays the release of progeny virions from the surface of infected cells [62], suppressing the viral population, thus allowing time for the host immune system to eliminate the virus.

Antigenic differences are used to classify influenza type A viruses into nine NA (N1–N9) subtypes [63]. Phylogenetically, there are two groups of NAs: group 1 contains N1, N4, N5 and N8, and group 2 contains N2, N3, N6, N7 and N9 [64].

In both influenza A and B, functional NA is a tetramer of identical subunits with four-fold rotational symmetry. The NA tetramer forms a box-like head on top of a long stalk domain and is anchored in the viral membrane by a hydrophobic sequence near the N-terminus [65,66]. The surface of an influenza virus typically has about 50 tetrameric NA spikes [67]. NA has been targeted in structure-based enzyme inhibitor design programs that have resulted in the production of two drugs, zanamivir (Relenza) [68] and oseltamivir (Tamiflu) [69], that mimic the transition state of the normal enzyme reaction.

**Figure 7:**
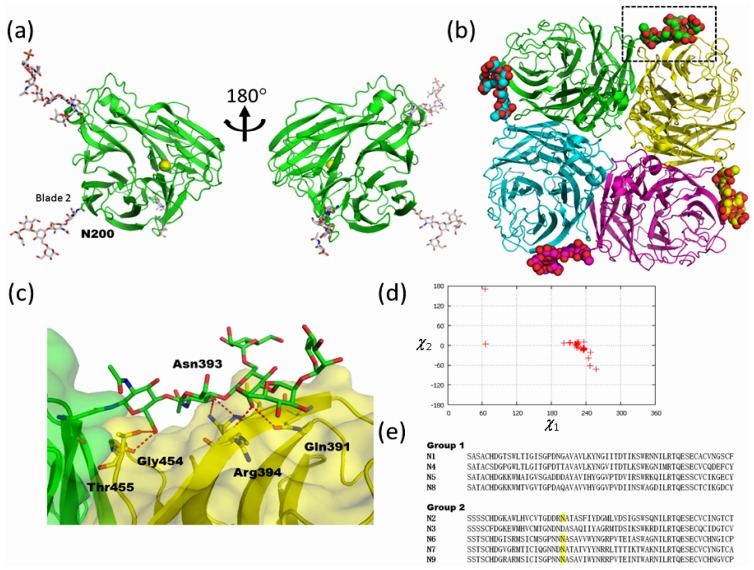
High-mannose type glycan of influenza neuraminidase assists tetramer formation. (**a**) Overall structure of monomeric influenza N2 neuraminidase (PDB code; 1nn2). Protein, carbohydrate, and calcium ion are shown in ribbon, stick, and sphere models, respectively. (**b**) Tetrameric structure of influenza N2 neuraminidase (PDB code; 1nn2). *N*-linked glycans at Asn200 are shown in sphere models. The region delineated in black dotted lines is magnified in (c). (**c**) Close-up view of *N*-glycan at Asn200 and symmetry related molecule. Hydrogen bonds are shown in red dotted lines. (**d**) The side-chain torsion angles of Asn200 of N2, Asn207 of N6, and Asn200 of N9 NA (Asn201 in PDB code; 2b8h). (**e**) Amino acid sequence alignment of group 1 and 2 influenza neuraminidase around Asn200 glycosylation sites. Putative *N*-linked glycosylation sites in group 2 are highlighted.

Crystallographic analysis of influenza NA has a history of almost 30 years. The first crystal structure of influenza N2 NA was reported in 1983 [70], and the first N9 in 1987 [71]. The NA monomer consists of a symmetric six-bladed β-propeller arrangement stabilized in part by calcium ions bound on the symmetry axis (Figure 7a). There are five *N*-glycosylation sequons exposed on the protein surface, namely Asn86, Asn146, Asn200, Asn234, and Asn402, the first four of which are assigned in the model. The two *N*-glycans at Asn146 and Asn200 are highly ordered. The glycan at Asn146 is of the core-fucosylated complex type, whereas that at Asn200 is of the high-mannose type. The catalytic active site is in the middle of the β-propeller. In the tetrameric enzyme, each active site is directed sideways rather than upwards, an orientation consistent with the enzyme having to cleave off sialic acid from nearby membrane proteins to avoid virus trapping (Figure 7b). The tetramer is approximately 100 × 100 × 60 Å in size with a large hole underneath around the 4-fold axis. The high-mannose type glycan at Asn200 is located in the rim of blade 2 and bridges the right-handed neighboring subunit, contributing to the intersubunit interactions in the tetramer (Figure 7b). A close up view of the *N*-glycan attached at Asn200 and neighboring molecules is shown in Figure 7c. The *N*-glycan lies over blade 5 of a neighboring molecule. A β-Man (Man-3) residue of this glycan is buried in the neighboring molecule. The accessible surface area of this residue is calculated as ~19 Å^2^ by AREAIMOL [72]. The crystal structures of N2, N6 and N9 NA in group 2 have been determined and are summarized in Table 2. The *N*-glycan attached at Asn200 in N2 corresponds to those of Asn207 in N6 and Asn200 in N9 and their structures are well superimposed on each other. The distribution of the torsion angles of the side chain of Asn200 is shown in Figure 7d. The *χ*1 torsion angles mainly assume ~240°, which is normally rare in both glycosylated and non-glycosylated Asn side chain conformers [48] (Figure 7d). Strong interaction between the *N*-glycan and the neighboring subunit may stabilize such a conformation of Asn200. Amino acid sequence alignment reveals the sequon at this site to be conserved among group 2 NAs except for N3 (Figure 7e). Structural comparison between group 2 and other NAs (N1, N4, N8 and type B NAs) reveal no obvious common structural feature [64,73]. Viral proteins are synthesized and secreted by host cells. Thus, the glycans attached on viral proteins are also processed by host glycosyl-hydrolases and -transferases. Since glycan structures of Asn200 are of the high-mannose type, group 2 NA likely forms a tetrameric structure before glycan processing.

## 3. Immature High-Mannose Type Glycans Contribute to Inter-Subunit and Inter-Domain Interactions

Initial processing of glycoproteins takes the form of deglucosylation of high-mannose type glycan in the ER, and is conserved among all eukaryotes [80]. It is generally considered to be the major event to signal the completion of protein folding. Mono- or di-glucosylated *N*-glycans are rarely observed in mature glycoproteins. However, glucosylated *N*-glycans have been detected in several secreted proteins. Here, we introduce two structures which possess immature glucosylated high-mannose type *N*-glycans on their surfaces. In both cases, the immature glycan extensively interacts with the surface of the protein. Investigation of the co-existence of both unprocessed and processed glycans on a single polypeptide may help to unravel the relationship between protein folding and glycan maturation.

### 3.1. Monoglucosylated High-Mannose Type Glycan Stabilizes Hexamer Formation of Arylphorin from *Antheraea pernyi*

Our first example is arylphorin from the Chinese oak silkworm, *Antheraea pernyi* (abbreviated as APA hereafter), which is a hexameric protein of 688 amino acid residues per subunit [81]. It is a hexamerin, a group of proteins belonging to a superfamily that includes arthropod tyrosinase, arthropod hemocyanin, and dipteran arylphorin receptor [82]. The hexamerins show clear structural similarities with the hemocyanins, but have lost the ability to bind copper ions and transport oxygen. They are synthesized in the fat body of a wide range of lepidopteran and dipteran larvae, among other insect orders. These proteins accumulate to high concentrations in the hemolymph. Hexamerins appear to serve as a storage form of amino acids, a resource required for complete development of the adult, since insect pupae do not feed during metamorphosis. In addition to being a storage protein, hexamerins appear to play other important roles during the lifespan of insects. There are at least two types of hexamerins in Lepidoptera: arylphorin and a methionine-rich storage protein [83].

**Figure 8:**
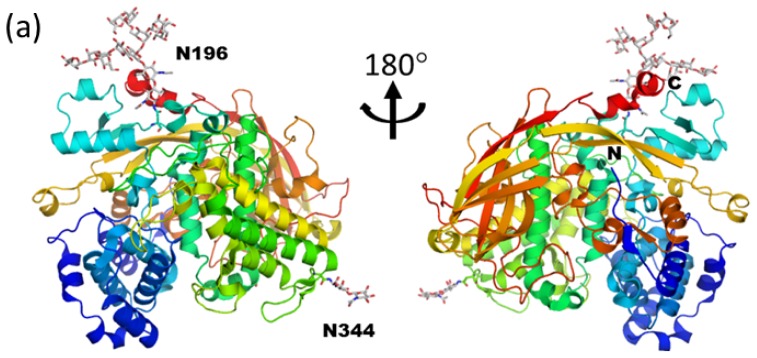

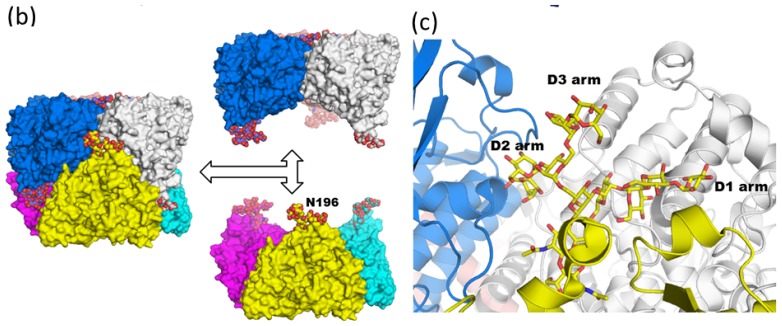
Immature monoglucosylated *N*-glycan on *Antheraea pernyi* arylphorin (PDB code; 3gwj) (**a**) Overall structure of monomeric APA. Protein and carbohydrate are shown in ribbon and rod models, respectively. (**b**) Hexameric structure of APA. Each monomer is shown in surface model. The attached *N*-linked glycans at Asn196 are shown in spheres. (**c**) Close-up view of Asn196-attached *N*-glycan in hexameric APA.

APA has two *N*-glycans attached at Asn196 and Asn344, although APA possesses four possible candidate sequons. Glycosylation of Asn344 is critical for the folding process, whereas glycosylation of Asn196 is not. Mass spectrometric analysis revealed that N344-glycan is a trimmed high-mannose type (Man_5-6_GlcNAc_2_), whereas the N196-glycan remains in a monoglucosylated *N*-glycan (Glc_1_Man_9_GlcNAc_2_) state and is resistant to peptide *N*-glycosidase F (PNGaseF) treatment [84]. Although the recombinant N344Q mutant protein is not secreted in culture medium, the Asn196Gln mutant protein is expressed as in wild type and has the same ecdysone-binding activity as wild-type. The crystal structure of APA was solved at 2.42 Å resolution. The overall structure of APA is similar to that of lobster hemocyanin and is composed of an N-terminal all α-helical fold and a C-terminal β-sandwich like fold (Figure 8a). The *N*-glycan at Asn344 is exposed to solvent and only the chitobiose portion is assigned. In contrast, the *N*-glycan at Asn196 has a clear electron density map and is assigned as a monoglucosylated structure. The asymmetric unit of the APA crystal contains six monomers (one hexamer) as shown in Figure 8b. While all *N*-glycan chains at Asn344 are completely exposed to solvent in the hexamer, the *N*-glycans at Asn196 are buried inside the hexamer and well organized in the deep cleft of the subunit interface. A comparison between monomeric and hexameric APA revealed that the D1 arms of the *N*-glycans are buried inside during hexamer formation. Actually, the accessible surface areas of a D1 arm in monomeric and hexameric APA are 370, and 195 Å^2^, respectively. The *N*-glycan forms about 20 direct water-mediated hydrogen bonds with adjacent amino acid residues, which are located in the same or different subunits. Typical dihedral *φ* and *ψ* angles at Manα1-2Man are ~60° and ~150°, respectively. In contrast, the dihedral *φ* and *ψ* angles at Manα1-2Man in the D2 arm (Man-D2-Man-A) are 279° and 130°, respectively. The D2 arm of the glycan adopts a unique conformation to accommodate the curvature formed by the blue and yellow molecules (Figure 8c). Indeed, the accessible surface of area of α1-2 linked Man in the D2 arm (Man-D2) is dramatically different in monomeric (217 Å^2^) and hexameric (86 Å^2^) APA structures. In addition to the inter-subunit disulfide bond between Cys73 and Cys649, extensive intermolecular interactions between *N*-glycan and APA also stabilize the overall trimer-trimer interaction by enhancing interaction between each top and bottom dimer (Figure 8b). Analytical ultracentrifugation and guanidinium chloride unfolding experiments revealed that the presence of the N196-glycan is important for stabilizing the hexameric state and overall stability of APA.

### 3.2. Diglucosylated *N*-Glycan Stabilizes Inter-Domain Interaction of β-Dalactosidase from *Trichoderma reesei*

β-galactosidase is an enzyme (E.C. 3.2.1.23) that catalyzes the hydrolysis of β1-3 or β1-4 linked Gal residues in oligo- and disaccharides, such as lactose, galactobiose, aryl- and alkyl-β-D-galactosides. This enzyme also has the ability to catalyze the reverse reaction of the hydrolysis called transglycosylation. β-galactosidases have been isolated from various sources, such as animals, plants, bacteria, yeasts and fungi. They have many important applications in the industrial and biotechnological fields. In the CAZy database [85], the β-galactosidases occur in the GH 1, 2, 35, and 42 subfamilies.

**Figure 9:**
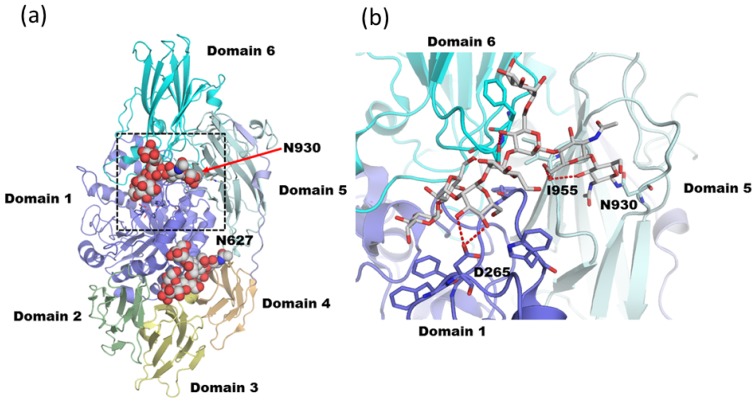
Immature diglucosylated *N*-glycan on β-galactosidase from *Trichoderma reesei* (**a**) Overall structure of Tr-β-gal (PDB code; 3ogv). *N*-linked glycan at Asn627 and Asn930 are shown in sphere models. The region delineated in black dotted lines is magnified in (b). (**b**) Close-up view of Asn930 attached glycan. Hydrogen bonds are shown as red dotted lines.

*Trichoderma reesei* β-galactosidase (Tr-β-gal) belongs to the GH 35 subfamily. The sequence and the enzymatic properties of this industrially useful enzyme have previously been reported [86,87]. Crystal structures of *Trichoderma reesei* β-galactosidase (Tr-β-gal) in unliganded and ligand complexes were solved at 1.2~1.75 Å resolutions (PDB code; 3og2, 3ogr, 3ogs and 3ogv [88]). The overall structure of Tr-β-gal consists of six domains (Figure 9a). The N-terminal domain forms an eight-stranded α/β barrel structure and is responsible for the catalytic reaction. The subsequent five domains form anti-parallel β-sandwich structures. Tr-β-gal possesses 11 putative *N*-linked glycosylation sites on the surface of the protein [87]. Ten of the 11 sites are exposed (Asn810 is buried). Electron density maps corresponding to the carbohydrate moieties of the *N*-glycans are observed at five of the positions. Two of these (Asn627 and Asn930) contain oligosaccharide chains that represent high-mannose glycan forms. One of these positions (Asn930) has diglucosylated high-mannose type glycan of the form Glc_2_Man_8_GlcNAc_2_ (PDB code; 3og2). The glycan at Asn930 is located between three different domains (first, fifth, and sixth domains) and makes a number of hydrogen bonds with the protein surface, stabilizing the structure of Tr-β-gal (Figure 9b). The carbonyl oxygen from Ile955 interacts with the chitobiose core. The side chain of Asp776 bridges β-Man (Man-3) and Glc residues. The glycan at Asn930 is also connected to the catalytic domain because OD1 and OD2 from Asp265 tightly interact with an α1-2 linked Man (Man-C). This glycan covers several hydrophobic and aromatic amino acid residues and may protect the structure from proteolysis. The β-Man (Man-3) is the most buried residue and reaches to the protein cavity (accessible surface area only ~36 Å^2^). The glucose units of Tr-β-gal reach very close to the catalytic site. Thus, it is plausible that glycosylation affects the catalytic properties of the enzyme.

Recombinant Tr-β-gal was overexpressed by a *Trichoderma reesei* expression system [89]. In fungal expression systems, Glc residues are expected to be trimmed by glucosidases I (GLS-I) and II (GLS-II). Nevertheless the *N*-glycan attached at Asn930 in Tr-β-gal is di-glucosylated and interacts with residues far apart in the primary sequence. It suggests that the protein folding of Tr-β-gal might be finished before GLS-II encounters the terminal Glc residues on the glycan. The tight association between the D1 arm and the protein surface likely prevents access of the glucose units to the catalytic site of GLS-II, inhibiting deglucosylation and further conversion to complex-type glycans.

## 4. What Is the Function of Mobile/Disordered *N*-Glycans?

Cell surface membrane proteins, such as cell surface receptors, are often glycosylated. Crystal structures of these cell surface glycoproteins revealed that their *N*-glycans are usually mobile and most of the sugars disordered. Although surface *N*-glycans are sometimes immobilized by symmetry-related molecules in crystal packing, only the first one or two GlcNAc residues are usually ordered enough to be traced in the electron density map. The alteration of glycoform is associated with various physiological and pathological events, including tumor invasion [90]. These disordered or “missing” glycans are therefore considered to assume certain physiological functions as with the highly-ordered *N*-glycans. In this chapter, we introduce several structures of cell surface glycoproteins which play a role in the immune system and cell-cell adhesion.

**Figure 10:**
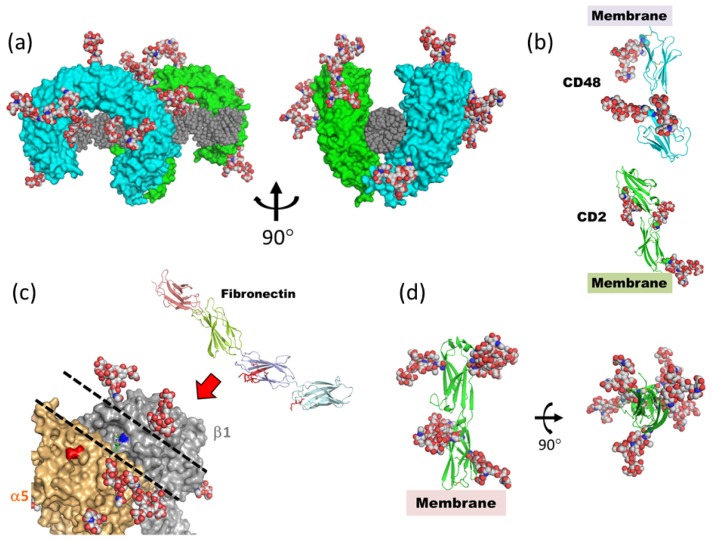
Highly flexible *N*-glycans on cell surface receptors. Complex-type *N*-glycans (GlcNAc_2_Man_3_GlcNAc_2_Fuc) are superimposed, based on the position of chitobiose or sequons by using LSQKAB [103]. (**a**) Fully glycosylated Toll-like receptor-3 (TLR3) ectodomain in complex with dsRNA (PDB code; 3ciy). Protein molecules are shown as green and cyan surface models. dsRNA is shown as a gray sphere. (**b**) Extracellular domains of CD2 (PDB code; 1hnf) and CD48 (PDB code; 2dru). (**c**) Crystal structure of α5β1 integrin ectodomain (PDB code; 3vi4) and fibronectin FN7-10 fragment (PDB code; 1fnf). In the fibronectin structure, the amino acid residues which interact with α5β1 integrin are shown in red stick model. Dashed lines on α5β1 integrin outline the shallow groove formed by *N*-glycans. (**d**) Intercellular cell adhesion molecule (ICAM)-2 ectodomains (PDB code; 1zxq).

Infectious diseases caused by various pathogens account for about one-third of all human deaths in the world, more than all forms of cancer combined [91]. To fight against these powerful pathogens, vertebrates use two types of immune defense carried out by specialized proteins and cells; the innate immune response and the adaptive immune response. Innate immunity is based on an ancient and ubiquitous system of cells and molecules that defend the host against infection. This system can recognize virtually all microbes using a limited repertoire of germ-line-encoded receptors that recognize broadly conserved components of bacterial and fungal cell walls or genetic material, such as double-stranded viral RNA (dsRNA) [92,93]. Toll-like receptors (TLRs) are the most important sensors in the innate immune system [94]. Ten human TLRs (TLR1~10) which specifically recognize pathogen-associated molecules have been identified. Human TLR3 is activated by dsRNA associated with viral infection, endogenous cellular mRNA, and sequence-independent small interfering RNAs. The human TLR3 ectodomain is a large horseshoe-shaped solenoid-like structure assembled from 23 leucine-rich repeats (PDB code; 1ziw, [95]). Human TLR3 ectodomain possesses 15 potential *N*-glycosylation sites. Due to the poor electron density of the carbohydrate moieties, only one or two GlcNAc residues are assigned at eight of the *N*-glycosylation sites. A putative fully glycosylated model structure reveals that almost all the surface of the TLR3 molecule is covered by carbohydrate, but one face is glycosylation-free. The structure of mouse TLR3 ectodomain in complex with dsRNA demonstrates that dsRNA contacts occur through residues on the glycosylation-free surface (PDB code; 3ciy, [96]). The glycosylation sites of both human and mouse TLR3 are almost identical. Compared with human TLR3, the electron density of carbohydrate is clearly observed in mouse TLR3. *N*-glycan at Asn413, located in the vicinity of dsRNA, is assigned as Man_3_GlcNAc_2_ and an α1-6 linked Man residue interacts with the sugar-phosphate backbone of dsRNA. As in the case of human TLR3, the location of *N*-glycan is thought to be useful in order to restrict the preferential orientation of ligand (Figure 10a).

As for adaptive immune systems, most of the cell surface receptors which are involved in antigen recognition by T cells and in the orchestration of the subsequent cell signaling events are glycosylated. Rudd *et al.* postulated the *N*-glycans on the protein surface play a wide range of roles, such as controlling the assembly and stabilization of the protein complexes in the adaptive immune system [97]. *N*-linked glycans attached on the membrane proximal domains of CD2 (PDB code; 1hnf, [98]) and CD48 (PDB code; 2dru, [99]) are distributed so as to provide a scaffold to orient the binding faces, which leads to increased apparent affinity (Figure 10b). Moreover, the glycans on T-cell receptors (TCR) are located over the protein surface in such a way that they could prevent non-specific aggregation. Another important point to be emphasized is that *N*-glycans limit the possible geometry and spacing of TCR/major histocompatibility complex (MHC) clusters which precede cell signaling.

Cells produce, organize and degrade extracellular matrix. The matrix in turn exerts a powerful influence on the cells, mainly through “matrix receptors”. Integrins are the principle matrix receptors on animal cells and transmit bidirectional signals across the plasma membrane, thereby linking the extracellular environment to the internal actin cytoskeleton [100]. All integrins are non-covalently linked heterodimeric molecules consisting of one α and one β subunit, which together create a binding site for specific extracellular ligands on that part of the protein furthest from the membrane. α5β1 integrin is a major cellular receptor for the extracellular matrix protein fibronectin and plays a fundamental role during mammalian development. Fibronectin is a principle component of the extracellular matrix and is a modular protein composed of homologous repeats of small domains with an elongated shape arranged as “beads on a string”. α5β1 integrin interacts with fibronectin through Arg-Gly-Asp (RGD) sequences present in a flexible loop region in the middle of the protein. A crystal structure of the α5β1 integrin ectodomain shows the fibronectin-binding pocket surrounded by four *N*-glycans (two in α5 and the other two in β1), forming a trench-like exposed surface along the subunit interface. This topography and location of the *N*-glycans presumably limits the choice of docking orientations when the elongated fibronectin molecule tries to make close contact (Docking model based on α5β1 integrin ectodomain (PDB code; 3vi4) and fibronectin fragment (PDB code; 2mfn and 1fnf), [101,102] (Figure 10c).

The intercellular adhesion molecules 1~3 (ICAM-1~3) are members of the immunoglobulin superfamily (IgSF) and are known ligands for integrins on the surface of cells. ICAM-2, a ligand for αLβ2 integrin, is composed of two *N*-terminal extracellular Ig domains (which share 35% sequence homology with ICAM-1), a transmembrane domain, and a short cytoplasmic tail. The Ig domain, the characteristic building block of IgSF, consists of two antiparallel β-sheets packed tightly against each other and linked by a disulfide bond. The crystal structure of the two extracellular Ig domains of ICAM-2 (PDB code: 1zxq) shows that they adopt a hockey stick shape. Six *N*-glycans are exposed to solvent and assigned as chitobiose portions. When one looks down the axis of the two Ig domains, these *N*-glycans are seen as uniformly distributed around the perimeter of the domains [104] (Figure 10d), and are well placed to prevent non-specific protein aggregation.

These examples illustrate the important role of glycan in assisting the docking of large ligands, both by orienting the ligand and by spacing the receptor through inhibiting aggregation. It has been considered that a principle role of the mobile carbohydrate attached on cell surface receptors is to increase protein conformational stability [105]. Thus solution analysis of RNaseB revealed that the glycan has a significant stabilizing effect on the protein structure by decreasing the flexibility of the protein backbone both near to and distant from the glycan attachment site [106]. These secondary functions of glycans, supplementary to actual binding interactions, seem to be fundamental to the role of the carbohydrates in glycoproteins on cell surfaces.

## 5. Future Perspective

Several structural and functional aspects of glycosylation, in terms of intra- and inter- molecular interactions, are provided by available crystal structures. An emphasis on glycoprotein-oriented structural biology will further the understanding of glycan function. Crystallographic analysis of glycoproteins will require a more thorough investigation of the glycoprotein expression system and of glycan structure. Although methodologies for glycoprotein production are advancing [107,108], improvements are required in the expression and selection of a particular glycoform of a target protein. The glycan sequence at each glycosylation site can be analyzed in advance by mass spectrometry, coupled with liquid chromatography. However, even with this sequence information, the electron density of the glycan must be interpreted with great caution. Backbone flexibility dictates that structural information on the glycan is largely missing in X-ray diffraction data. Other techniques, such as molecular dynamic simulation and NMR, are needed to help join the discontinuous snapshots derived from X-ray studies and to evaluate the contribution of the glycan moieties to molecular fluctuations.

The glycoform at a particular site on a protein is often closely related to physiological function and is tightly regulated [18,46,47,109]. However, in many cases the structural relationship between glycoform and function still remains unclear. Recent technical advances in the chemical and enzymatic syntheses of homogeneous glycoproteins are going to make further valuable contributions to the study of glycoform-function relationships [110–112]. A survey of current PDB data indicates that the glycan structures of available glycoproteins are mainly of the high-mannose type, and may be biased due to the expression system used. Thus the relationship between glycoform function and its 3D structure needs careful investigation. Structural biology focusing on glycoform-specific functions is the challenge for the near future.

## Figures and Tables

**Table 1:** Summary of Fc fragment structures.

PDB ID	Glycan structure *	Resolution	Reference
Human IgG1 Fc fragment produced by HEK293T cell + kifunensine			
2wah	M_7_GN_2_ (chain-A)	2.51	[30]
	M_1_GN_2_ (chain-B)	-	-
Human IgG1 Fc fragment by enzymatic treatment			
1h3t	M_1_GN_2_F	2.4	[31]
1h3u	M_3_GN_2_F	2.4	enzymatic
1h3x	GN_2_M_3_GN_2_F	2.44	treatment
1h3v	G_2_GN_2_M_3_GN_2_F	3.1	[32])
1h3w	G_2_GN_2_M_3_GN_2_F	2.82	-
1h3y	G_2_GN_2_M_3_GN_2_F	4.1	-
Human IgG1 Fc produced by CHO or Fut8^−/−^ CHO cells			
3ave (2dtq)	GN_2_M_3_GN_2_F	2.0	[29]
2dts	GN_1_M_3_GN_2_ (chain-A)	2.2	-
	GN_2_M_3_GN_2_ (chain-B)	-	-
Human IgG1 Fc fragment triple mutant (M252Y/S254T/T256E)			
3fjt	GN_2_M_3_GN_2_F	2.5	[33]
Human IgG1 Fc fragment triple mutant (L234F/L235E/P331S)			
3c2s	G_1_GN_2_M_3_GN_2_F	2.3	[34]
Protein is produced by HEK293 cells.			
Human IgG1 Fc fragment triple mutant (S239D/A330L/I332E)			
2ql1	G_1_GN_2_M_3_GN_2_F	2.5	[35]
Human IgG1 Fc fragment + 13 residues peptide			
1dn2	G_1_GN_1_M_3_GN_2_F (chain-A)	2.7	[36]
	G_1_GN_2_M_3_GN_2_F (chain-B)	-	-
Human IgG1 (Rituxan) Fc fragment + *Staphylococcus aureus* Protein A domain B			
1l6x	G_2_GN_2_M_3_GN_2_F	1.65	[37]
Human IgG1 Fc fragment			
3do3	G_1_GN_2_M_3_GN_2_F	2.5	[38]
Human IgG1			
1hzh	G_2_GN_2_M_3_GN_2_F (chain-H)	2.7	[39]
	G_1_GN_2_M_3_GN_2_F (chain-K)	-	-
Mouse IgG2a			
1igt	G_1_GN_2_M_3_GN_2_F	2.8	[19]
Human IgG1 Fc fragment + Protein-A mimetic peptide dendrimer.			
3d6g	GN_2_M_3_GN_2_F	2.30	[40]
Mouse IgG2b Fc fragment			
2rgs	GN_2_M_3_GN_2_F	2.1	[41]
Rabbit IgG Fc fragment			
2vuo	G_1_GN_2_M_3_GN_2_	1.95	[42]
Human IgG1 Fc fragment + minimized protein A			
1oqo	GN_2_M_3_GN_2_F	2.3	[43]
1oqx	M_3_GN_2_F	2.6	-
Human IgG Fc fragment			
1fc1	G_1_GN_2_M_3_GN_2_F	2.9	[17]
Rat IgG2a Fc fragment			
1i1c	GN_2_M_3_GN_2_F	2.7	[18]
Human IgG1 Fc fragment + human Fc receptor (FcγRIIIb)			
1t83 (1its)	G_1_GN_1_M_3_GN_2_F (chain-A)	3.0	[44]
	GN_2_M_3_GN_2_F (chain-B)	-	-
1t89 (1iix)	G_1_GN_1_M_3_GN_2_F (chain-A)	3.5	-
	GN_2_M_3_GN_2_F (chain-B)	-	-
Human IgG1 Fc fragment + human Fc receptor (FcγRIIIb)			
1e4k	G_1_GN_2_M_3_GN_2_F	3.2	[45]
Human IgG1 Fc fragment + human Fc receptor (FcγRIIIa)			
3sgj	GN_2_M_3_GN_2_F	2.2	[46]
3sgk	GN_3_M_3_GN_2_	2.4	-
Human IgG1 Fc fragment + human Fc receptor (FcγRIIIa)			
3ay4	G_1_GN_2_M_3_GN_2_	2.2	[47]
Human heterodimeric Fc + human neonatal FcR (FnRn)			
1i1a	GN_2_M_3_GN_2_F	2.8	[18]

* Glycan structures deposited in each PDB coordinate file are shown. G: d-galactose, GN: *N*-acetyl-d-glucosamine, M: d-mannose, F: l-fucose.

**Table 2:** Summary of group 2 influenza neuraminidase structure.

PDB ID	Glycan structure *	Resolution	Reference
N2 (A/Tokyo/3/1967)			
1nn2	M_4_GN_2_	2.20	[74]
N2 (A/Tokyo/3/1967)			
1inw	M_3_GN_2_	2.40	[75]
1inx	M_3_GN_2_	2.40	-
N6 (A/swine/KU/2/2001)			
1v0z	M_3-5_GN_2_	1.84	[76]
1w1x	M_1-5_GN_2_	2.00	-
1w20	M_1-6_GN_2_	2.08	-
1w21	M_1-6_GN_2_	2.08	-
2cml	M_6_GN_2_	2.15	-
N9 (A/Tern/Australia/G70C/75)			
1iny	M_5_GN_2_	2.40	[75]
N9 (A/Tern/Australia/G70C/1975 (H11N9))			
1f8b	M_5_GN_2_	1.80	[77]
1f8c	M_5_GN_2_	1.70	-
1f8d	M_5_GN_2_	1.40	-
1f8e	M_5_GN_2_	1.40	-
N9 (A/Tern/Australia/G70C/1975) in complex with single chain Fv fragment			
1a14	M_5_GN_2_	2.50	[78]
N9 (A/NWS/whale/Maine/1/84)			
2b8h	M_7-8_GN_2_	2.20	[79]

* Glycan structures deposited in each PDB coordinate file are shown. M: d-mannose, GN: *N*-acetyl-d-glucosamine.

## Abbreviations

GlcNAc: *N*-acetyl-d-glucosamine
Man: d-mannose
Gal: d-galactose
Sia: sialic acid
Glc: d-glucose
Fuc: l-fucose
GnT: *N*-acetylglucosaminyltransferase
CHO: Chinese hamster ovary
HEK: human embryonic kidney
IgG: immunoglobulin G
Fc: crystallizable fragment
ADCC: antibody-dependent cellular cytotoxicity
FcRn: neonatal Fc receptor
NA: neuraminidase
APA: *Antheraea pernyi* arylphorin
Tr-β-gal: *Trichoderma reesei* β-galactosidase
TLR: Toll-like receptor
ICAM: intracellular adhesion molecule
